# Regulatory Science Needs for Neonates: A Call for Neonatal Community Collaboration and Innovation

**DOI:** 10.3389/fped.2014.00135

**Published:** 2014-12-01

**Authors:** Susan K. McCune, Yeruk Ager Mulugeta

**Affiliations:** ^1^Office of Translational Sciences, Center for Drug Evaluation and Research, U.S. Food and Drug Administration, Silver Spring, MD, USA; ^2^Office of Clinical Pharmacology, Office of Translational Sciences, Center for Drug Evaluation and Research, U.S. Food and Drug Administration, Silver Spring, MD, USA

**Keywords:** neonatal, regulatory science, drugs, trials, collaboration

Neonates have long been a vulnerable population to unstudied therapies, starting as early as the late nineteenth century with Mrs. Winslow’s Soothing Syrup, a purported remedy for teething that contained alcohol and morphine sulfate, causing numerous infant deaths (1). In 1959, Gray baby syndrome was first reported in infants receiving large doses of chloramphenicol as a broad spectrum antibacterial agent (2), and in 1983, Mulhall et al. (3) noted the altered pharmacokinetics (PK) of chloramphenicol in neonates and young infants resulting in elevated serum levels and some toxic effects.

During the last 50 years, we have witnessed amazing progress in neonatal care resulting in increased survival and improved outcomes for 23–24 weeks preterm infants and decreased morbidity and mortality associated with many neonatal diseases including primary pulmonary hypertension of the newborn (PPHN) and hypoxic ischemic encephalopathy (HIE). This progress has been buttressed by the development of therapies such as surfactant, nitric oxide, caffeine, and the Cool Cap, all of which are FDA labeled for use in the neonatal population. However, neonates are still exposed to a significant number of off-label medications during their hospitalization. Warrier et al. (4) reported that infants <28 weeks gestation were exposed to a mean of almost 12 drugs during their hospitalization, and Conroy and McIntyre (5) demonstrated that 93% of babies received at least one unlicensed or off-label medication during their hospitalization. A 2014 American Academy of Pediatrics Policy Statement acknowledged that off-label use of drugs, “presents an even larger and more complex issue in preterm and full term neonates” (6). Exposure to off-label drugs is only one side of the coin, as the subspecialty continues to lack sufficient new therapeutic interventions to treat most of the neonatal specific diseases.

Legislative mandates have encouraged studies in pediatric populations. Congress passed pediatric legislation under the Food and Drug Administration Modernization Act of 1997, which was followed by the Best Pharmaceuticals for Children Act (BPCA) in 2002 and the Pediatric Research Equity Act (PREA) in 2003. Both BPCA and PREA were made permanent under the Food and Drug Administration Safety and Innovation Act (FDASIA) in 2012 (7). A number of studies submitted in response to the pediatric legislative initiatives have included neonatal information for sedation, gastroesophageal reflux disease, drugs used in surgery (adjunct to general anesthesia, reversal of non-depolarizing neuromuscular agents), thrombosis with systemic to pulmonary artery shunts, head lice (safety information related to benzyl alcohol exposure), prevention of bronchopulmonary dysplasia (BPD), treatment of mild/moderate pain or fever, and HIV infection (8).

Despite efforts promoted by the legislation, the majority of these studies were not able to establish efficacy in the neonatal population. It is not clear whether the lack of efficacy is accurate or is related to study design issues including the study population, disease characteristics in the neonate, drug properties (dose, tissue effect, metabolism), or the inability of the endpoints to reflect clinically meaningful outcomes. Each of these areas needs to be addressed in future studies.

Future studies will need to carefully define the study populations. In an effort to maximize the number of study participants, many studies in preterm infants have included all infants born at <32 weeks gestation. A 4-week-old infant that was born at 24 weeks gestation is not the same as a 1-day-old born at 28 weeks gestation. The normal ontogenic processes *in utero* are disrupted by birth and postnatal care including exposure to a relatively hyperoxic environment (even in room air) with major changes in organ perfusion and alterations in nutrient/mineral delivery. As an example, it is not possible to achieve third trimester rates of calcium accretion in the preterm infant (9). A more precise definition of the inclusion criteria for studies, taking into consideration both gestational age and postnatal age, will potentially identify more homogeneous groups for studies and may be more accurate in the findings of safety and efficacy because the populations will be more appropriately matched.

Disease characteristics in the neonatal population are dependent on the gestational age at birth and the postnatal age of the infant. Surfactant has been shown to be a very effective therapy for respiratory distress syndrome but the degree of response may differ between gestational ages based on the development of the pulmonary architecture (10, 11). It is critically important to understand the pathophysiology of neonatal disease processes, not just at the time of birth but with respect to postnatal adaptive responses. This is especially true with respect to safety and understanding potential off-target effects of drugs in the developing neonate.

There are a number of examples of altered PK for drugs used in neonates including chloramphenicol (3), gentamicin (12), and most recently clindamycin (13). Drug elimination may even be faster in small preterm neonates and the dose requirement may be as much as triple that of the adults as seen in the publication on micafungin (14). It will be imperative to understand receptor and enzyme system ontogeny as well as perturbations due to postnatal changes when infants are born at various gestational ages from 24 weeks to full term. This has implications for absorption, distribution, metabolism, and excretion of drugs and is critically important for individual drug dosing but also for the development of physiologically based pharmacokinetic (PBPK) models to enhance optimal drug dosing for neonates. Formulation issues continue to plague neonatal drugs and will need to be addressed to ensure consistency in drug delivery and exposure.

Clinical trials are challenging in the neonatal population and will require innovative approaches leveraged from the rare disease experience (15). These efforts may include the use of master protocols (16) with innovative adaptive designs using a limited number of doses that are based on modeling and simulation efforts derived from data obtained through opportunistic sampling. Safety monitoring must be a priority and will need to be differentiated from confounding disease processes and concomitant medication exposure. Clinically meaningful endpoints will need to be developed by the neonatal community, and predictive and prognostic biomarkers should be developed to enrich trial populations. In 2010, the Center for Drug Evaluation and Research (CDER) established the Drug Development Tools (DDT) Qualification programs as part of the Critical Path Initiative to provide a framework for development and regulatory acceptance of scientific tools, including biomarkers, animal models for use under the Animal Rule, and clinical outcome assessments for use in drug development. The qualification decision will be made publicly available and qualified DDTs will allow drug developers to use the DDT in the qualified context of use without requesting that CDER reconsider and reconfirm the suitability of the DDT for the qualified context of use. Carefully designed biomarkers and endpoints will be the hallmark of studies for neonatal diseases (7). These novel approaches to neonatal clinical trials should be highlighted by current training programs.

As an adjunct to these clinical trial questions, it is critical that the data from these trials be in a format that permits cross-study analyses. In addition, enormous quantities of information are accrued on a daily basis on infants in the NICU. Some of this is captured in registries and some in electronic health records. These data may not be represented in a standardized way so that analyses are not possible across systems. This wealth of information from clinical trials, registries, and electronic health records should be mined to optimize study designs and to capture safety information but needs to be transformed and standardized to maximize comparative efforts.

As John Donne coined, “No Man is an Island” and these issues cannot be tackled by individuals alone. The neonatal community needs to work collaboratively to accomplish these difficult tasks. FDA’s Critical Path Initiative was launched in 2004, with the release of the report entitled, “Innovation/Stagnation: Challenge and Opportunity on the Critical Path to New Medical Products.” The report challenged the scientific community to work together to bridge scientific gaps, enhance translational sciences, and improve the medical product development process. Under the auspices of the Critical Path Initiative, FDA has collaborated through Public Private Partnerships (PPPs) with multiple stakeholders, including other Federal, academic, scientific, patient advocacy, and industry organizations, to facilitate the development of new tools to foster innovation in FDA-regulated product development. These PPPs enable stakeholders to leverage expertise and resources to conduct mutually beneficial research activities resulting in publicly available information. The public health need to protect neonates by affording them access to safe and efficacious therapies provides the basis for the development of a neonatal consortium to address all of these complex issues.

It is imperative that the neonatal community work collaboratively to develop and label drugs for neonatal diseases. Basic science research, critical to defining the pathophysiology of disease and the ontogeny of metabolic pathways, provides the infrastructure for innovative approaches to studying drugs in neonates. The basic science supports modeling and simulation efforts to establish optimal dosage strategies and promotes the development of clinically meaningful short-term and long-term endpoints that can be implemented in innovative trial strategies. It is critically important that all sources of information be leveraged and standardized in this effort including data from clinical trials, registries, and electronic health records. A neonatal consortium effort would create a forum to bring academic, industry, patient advocacy, and government stakeholders together to consolidate the approach to maximize opportunities to label drugs for use in neonates. The initial discussion of a potential neonatal consortium, co-sponsored by the Critical Path Institute, Burroughs-Wellcome Fund, and FDA took place on the FDA White Oak campus on October 28 and 29, 2014. Hopefully, this will launch a productive collaborative effort to spur drug development for neonates.

## Conflict of Interest Statement

The authors declare that the research was conducted in the absence of any commercial or financial relationships that could be construed as a potential conflict of interest.
